# Effects of HIIT and MIIT Suspension Training Programs on Sleep Quality and Fatigue in Older Adults: Randomized Controlled Clinical Trial

**DOI:** 10.3390/ijerph18031211

**Published:** 2021-01-29

**Authors:** José Daniel Jiménez-García, Fidel Hita-Contreras, Manuel Jesús de la Torre-Cruz, Agustín Aibar-Almazán, Alexander Achalandabaso-Ochoa, Raquel Fábrega-Cuadros, Antonio Martínez-Amat

**Affiliations:** 1 MOVE-IT Research Group, Department of Physical Education, Faculty of Education Sciences, University of Cádiz, 11003 Cádiz, Spain; josedaniel.jimenez@uca.es; 2 Department of Physical Education, Faculty of Education Sciences, University of Cádiz, 11003 Cádiz, Spain; 3 Department of Health Sciences, Faculty of Health Sciences, University of Jaén, 23071 Jaén, Spain; fhita@ujaen.es (F.H.-C.); aaochoa@ujaen.es (A.A.-O.); rfabrega@ujaen.es (R.F.-C.); amamat@ujaen.es (A.M.-A.); 4 Department of Psychology, University of Jaén, E-23071 Jaén, Spain; majecruz@ujaen.es

**Keywords:** HIIT, TRX, older adults, sleep quality, fatigue

## Abstract

Poor sleep quality lessens general health quality and is related to physical and mental problems. Moreover, fatigue is one of the foremost common complaints in medical care and plays a role in the decreasing quality of life of the older population. For these reasons, the objective of this study was to examine the effect of high- and moderate-intensity interval training programs (HIIT vs. MIIT)—both consisting of twelve weeks of TRX training—on the sleep quality and fatigue levels of the elderly. A randomized controlled clinical trial (NCT03404830) was conducted. A total of 82 subjects were randomized to either a HIIT group (n = 28) that performed a main squat activity with a suspension system, comprising four four-minute intervals between 90–95% of the maximum heart rate (HR), an MIIT group (n = 27) with an intensity of 70% of the maximum HR, and a control group (CG) (n = 27) that continued their daily lifestyle. The two exercise groups trained twice a week for 12 weeks, with each session lasting 45 min. Sleep quality was measured using the Pittsburgh sleep quality index (PSQI), and fatigue was assessed using the fatigue severity scale (FSS). Outcomes were measured before the intervention and after the intervention period. Post-intervention sleep quality measurements revealed a statistically significant interaction regarding group × time (*p* < 0.005) and fatigue (*p* = 0.002). Specifically, fatigue decreased in the HIIT group between both measurement moments (*p* = 0.003). In addition, differences were obtained in the post-intervention measure between the HIIT and MIIT groups (*p* = 0.013) and HIIT and control (*p* = 0.029). Our analysis indicates that a population of the elderly showed improvements in sleep quality and fatigue after performing a high-intensity intervention using suspension training (TRX), with markedly better results in the HIIT group.

## 1. Introduction

In the last decades, the global population has experienced generalized aging, with the share of those aged 65 and over drastically increasing in our society [1]. This growth has an influence on frailty and how there are reliable tools that show differences between sex on frailty in the Spanish population [2,3]. In addition, current epidemiologic studies indicate that sleep disturbances are common among the elderly [4,5].

Sleep is essential for physical and mental well-being. Playing a fundamental role in the body’s maintenance, it is an essential requirement for life [6]. Poor sleep quality is a usual and increasing concern among the general population and is associated with several adverse effects on physical, mental and social well-being [7]. As age increases, sleep problems increase sharply [8]. It has been shown that during the aging process, a decrease in the duration of sleep occurs, generally associated with daytime sleepiness, daytime malfunction, and other health and safety problems [9]. In addition, sleep efficiency remains largely unchanged from infancy to adolescence but decreases significantly in adulthood [10], with grown-ups having shorter rapid eye movement sleep latencies, increasingly longer waking periods during sleep hours, and a lower awakening threshold [11]. There is also a greater number and duration of nocturnal awakenings. These age-related changes in sleep are related to normal physiological and psychosocial changes in aging [12]. Short sleep duration has also been linked to metabolic problems such as obesity in older people [13]. In a study of more than 80,000 healthy older people, those with short sleep duration showed an increase in weight and risk of developing obesity [14]. Moreover, poor sleep quality can have physical effects on the older population, including an increased risk of falls due to cognitive deficit and balance impairment [15], which affect both the mobility and independence of older people [16] and have a large impact on their quality of life. Moreover, short sleep duration and poor sleep efficiency have been associated with a higher risk of falling [17]. It has been well established that good sleep quality is related to healthy longevity [18]. Almost half of the older adult population complains of sleep problems, which are significantly related to morbidity and mortality in this population [19].

Fatigue is another of the most common complaints associated with poor sleep quality among older people [20]. In a study involving a group of older people who slept well and another who slept poorly, fatigue was significantly correlated with sleep duration, sleep efficiency, and time spent awake at night [21]. In turn, this relationship is associated with physical activity since lower levels of physical activity, and poor quality of sleep have been shown to be linked to symptoms of fatigue [22].

Physical activity is one of the most suitable low-cost tools for improving health given its proven multiorgan benefits [23]. Previous literature has shown the relationship between lower activity levels and decreased cognitive performance, physical deterioration, and mortality in older people [24]. Likewise, older adults who perform different types of physical activity have been shown to report significant improvements in sleep quality [25]. Moreover, a systematic review of randomized clinical trials has shown that interventions consisting of aerobic or strengthening exercises are more effective in improving sleep quality in older adults [26]. The most common limitation for performing physical activity is the lack of time [27]. To address this, a new approach to physical activity was developed, based on high-intensity interval training (HIIT) or short bouts of exercise at high-intensity. Generally speaking, HIIT is a type of exercise realized with intermittent high-intensity activity sessions combined with low-intensity or rest phases. Furthermore, it is perceived as a more satisfactory activity than continuous moderate-intensity training [28,29] and has been proven to be safe and well-tolerated in healthy subjects from different populations [29] and even in patients with pathologies such as heart disease [30]. In particular, an exercise program based on HIIT, receiving important attention in cardiac rehabilitation [31,32] and insulin resistance [33] in the elderly population.

Some of the studies mentioned above have suggested suspension training as a viable method to exercise [34]. Using one’s own weight as resistance allows for the intensity of each activity to be modified by altering the position of the body and its relationship with gravity. In any given exercise, the subject must grip the suspension training device, which has handles and two straps. Suspension training can be recommended for older adults since it improves muscle activation and balance [34]. To date, there have been very few studies involving suspension training in a population of older adults [34], but those that exist speak of its high degree of safety as a training system. An original research study [35] involving a 12-week training suspension program performed by 30 older women revealed improvements in physical fitness and percentage of body fat. Angleri et al. [36] concluded that suspension training is an alternative to resistance training as it allows for better control of several physical function variables among an older adult population. In terms of sleep quality, HIIT alone or combined with other types of workouts such as whole-body electrostimulation has been shown to have similar benefits in older people [37]. However, another study [38] found improvements in sleep quality in the groups doing moderate-intensity continuous training and stretching training, but not in the HIIT group. In contrast, a prospective randomized controlled trial that performed a 12-week intervention performed by testicular cancer survivors demonstrated the beneficial effects of HIIT on fatigue [39]. Furthermore, other studies realized in older adults showed improvements in functional capacity, balance, quality of life and cognitive status after squat exercise [40].

We hypothesized that a HIIT and moderate-intensity interval training (MIIT) performed with a suspension training system would improve sleep quality and fatigue of older adults after 12 weeks of training, and this benefit would be greater in the HIIT group.

For everything mentioned above, the objective of this study was to compare the effects that two 12-week TRX interval training programs (HIIT vs. MIIT) had on the sleep quality and fatigue of the elderly population.

## 2. Materials and Methods

This project was registered as NCT03404830 on clinicaltrials.gov (clinicaltrials.gov/ct2/show/NCT03404830); there may be some overlap in relation to general methodology and topics.

### 2.1. Study Design and Participants

The design of the present study is a randomized controlled clinical trial (RCT) that was part of a research work that tested the effects of two different intensities of TRX suspension interval exercises (HIIT and MIIT) on several health indicators in older adults in the community. The intervention took place from September to December 2018. The recruitment of subjects was performed through the municipal sports office of the town hall of Pizarra (Málaga, Spain), and all individuals were contacted through municipal registrations (by e-mail and telephone calls), local media and community networks. Initially, 90 people were contacted, and 82 complied with the inclusion criteria and voluntarily agreed to take part in this study. Inclusion criteria: aged > 60 years and being able to understand the instructions, protocols, programs of this research. Exclusion criteria: conditions that could affect the functional activity and balance (such as visual or vestibular dysfunctions) or that prevent the performance of physical tests, been under medication that may alter the balance, systematic diseases (i.e., cancer, diabetes mellitus, musculoskeletal conditions or heart disease), psychiatric or neurological pathologies, conditions or is already enrolled in another exercise program. Prior to the beginning of the intervention, all individuals gave their written informed consent. This study received the approval of the local Human Ethics Committee of the University of Jaén (DIC.17/5.TES), with the Declaration of Helsinki, good clinical practices, and applicable laws and regulations, and following the ethical standards in sport and exercise science research [41].

### 2.2. Sample Size Calculation

The Ene 3.0 software (GlaxoSmithKline, SA, Madrid, Spain) was used to assess the sample size. Using as reference the data of Akbari-Kamrani et al. [42], if a total of 12 experimental units are included, distributed among the 3 groups according to the proportions set by the researcher, a power of 95.00% can be achieved to detect differences in the contrast of the null hypothesis H_0_. The means of the 3 groups are the same using a 1-Factor ANOVA test for independent samples, taking into account that the level of significance is 5.00%, and assuming that the variability between groups is 1.24 and the variability within groups is 0.61.

### 2.3. Allocation to Intervention

**Figure 1:**
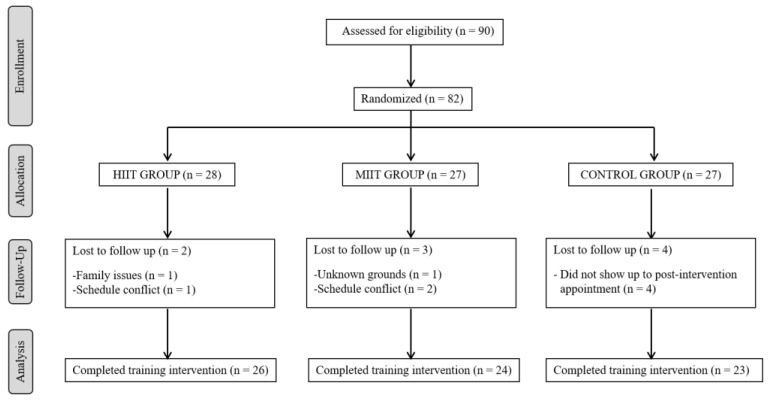
Flow diagram of study design.

Subjects included in the study were randomly assigned using a computer generating a table of random numbers, either to a HIIT group (HIIT), MIIT group (MIIT) or a control group (CONTROL) in a 1:1:1 ratio. Participants, sports instructors and investigators, were blinded to group assignment. Assignments were collected in an opaque envelope sealed in a sealed location and then opened by an independent party who did not assist in selecting the subject or evaluating outcomes or treatment. Initially, 28 individuals were assigned to the HIIT group, 27 to the MIIT group and 27 to the CG. Subsequently, the participants were reduced to 26 for HIIT (68.23 ± 2.77 years, 92.3% women), 24 for MIIT (68.75 ± 5.98 years, 70.8% women) and 23 for the CG (68.52 ± 6.33 years, 65.2% women) for different causes. Figure 1 shows a flow chart of participants consistent with the CONSORT statement extension for randomized controlled trials with non-pharmacological treatment [43]. The HIIT and MIIT groups administered a suspension training program for twelve weeks, and the CG individuals continued their daily lifestyle and were instructed to avoid any regular exercise and to participate in other exercise programs.

### 2.4. Procedures

Outcomes were assessed at baseline and immediately after the 12-week training period. Sociodemographic characteristics such as sex, age and smoking habits were collected at baseline. Weight was measured with a 100 g–130 kg precision digital weight scale (Tefal, Barcelona, Spain), and height was obtained with a T201-T4 Asimed height scale (Asimed T201-T4, Barcelona, Spain). Body mass index (BMI) was acquired by dividing weight by height (kg/m^2^) [44]. The independent variables were the HIIT TRX system training program and the MIIT TRX system training program, and the dependent variables were sleep quality and fatigue.

#### 2.4.1. Sleep Quality

Subjective sleep quality in the four previous weeks was measured with the Pittsburgh sleep quality index (PSQI) [23,45]. This tool consists of nineteen self-rated questions, as well as five other questions (only for clinical information) that must be answered by roommates. The PSQI produces a total score (ranging 0 to 21)) and seven different domain scores (ranging 0 to 3): sleep quality, sleep latency, sleep duration, sleep efficiency, sleep disturbances, use of sleep medication and daytime dysfunction. Greater scores indicate poorer sleep quality. A PSQI total >5 indicates poor sleep quality. Minimal important differences (MID) of ≥3 have been described for the PSQI total score.

#### 2.4.2. Fatigue

The fatigue severity scale (FSS) is one of the foremost usually employed self-report tools that assess fatigue [46,47]. It is composed of nine items (raging 1 to 7). The mean score of the nine items is employed the FSS total score. A higher score indicates greater severity of fatigue. MID ranges from 0.5 to 1.2 for the FSS total score.

### 2.5. Training Programs

The suspension training instrument was composed of two handles and two straps that need continuous grip. The exercise regimen proposed during this study was carried out using the TRX (Fitness Anywhere LLC, San Francisco, CA, USA). Prior to the exercises, subjects of the HIIT and MIIT groups familiarized themselves with the tool over a four-week period (two sessions per week with video expositions and six repetition attempts). Most of the training was done in the crouched position, and the straps were molded to medium length. Subjects began to stand up, spotting the anchor point, holding with elbows flexed at 90° and the handles with the whole hand. The subjects crouched and then rose to succeed in the starting position. For subjects assigned to the HIIT group, each session was structured in three stages: a warm-up (10 min), the main activity of the main squat with suspension system distributed in four intervals of a duration of four minutes and with an intensity of 90–95% of the utmost pulse continued with active rest intervals of three minutes at 50–70% (25 min) and finally, a cool-down period (10 min). Within the MIIT group, subjects carried out a similar protocol to HIIT, with an intensity of 70% of the utmost pulse main squat activity continued with active rest intervals at 50–55%. To control the maximum H, we took into account the instructions from Ellingsen et al. [48]. Maximum pulse was controlled by the formula, validated for the elderly population, MHR = 208.75–0.7 × age [49]. The exercise intensity was monitored by the Polar V 800 (Polar Electro, Oy, Kempele, Finland), confirming that their H was maintained over a period previously defined by the target H with reasonable or vigorous effort and constant during the procedure. The exercises were led and controlled by two teachers who specialized in sport. Each participant had a heart rate monitor (Polar V 800), and two sports instructors supervised the duration of each period of exercise in the main part, with subjects remaining in the H area determined for their assigned group (HIIT or MIIT). In addition, each participant had been previously provided with a leaflet containing information about training zones and their associated heart rate ranges so that they could have a clear idea of the level of effort and heart rate that their intervention group was expected to maintain for the longest possible time during each session. The two sports instructors supervised at all times that each participant remained in the target work zone. In turn, two physical therapists lent support and supervised the activity. The HIIT sessions were held on Tuesdays and Thursdays, while the MIIT sessions were held on Mondays and Wednesdays, at the same time (10:00–11:00 a.m.), which means that for each participant, at least a 24 h rest period was kept between sessions. Participants were eliminated if they missed more than five sessions in the intervention period, in which a record of injuries and other effects reported by the subjects.

### 2.6. Data Analysis

Continuous variables were described as mean and standard deviations, while categorical variables were presented as frequencies and percentages. Previously, Levene’s tests for equality of variances between groups did not show statistically significant results, *F*(2, 70) = 2.19, *p* = 0.120 (for the lowest FSS score). The chi-squared test and one-factor analysis of variance (ANOVA) were employed to analyze between-groups differences at baseline. A 3 × 2 mixed ANOVA was performed for each of the dependent variables (PSQI total score and domains and FSS total score), with the group as the between-groups variable (control group, MIIT group, or HIIT group) and the within-group variable being the measurement time (before and just after the intervention). The study of the possible interactions was performed using univariate ANOVA and Student’s *t-*tests for repeated measures. In the post-intervention measurements, two (subjective sleep quality) and three (daytime dysfunction) outlier values (values larger/higher than 2.5 standard deviations) were identified and not considered for the posterior analyses. Finally, effect sizes were interpreted using Cohen’s d statistic [50], where effect sizes were considered as negligible (Cohen’s d < 0.2), small (Cohen’s d ≥ 0.2 to ≤0.5), moderate (Cohen’s d ≥ 0.5 to ≤0.8) and large (Cohen’s d ≥ 0.8). SPSS v. 21.0 for Windows statistical software (SPSS Inc., Chicago, USA) was used for data analysis, a. The statistical significance level was set at a *p* value < 0.05.

## 3. Results

The average values for the group of participants in the anthropometric measurements of weight, height and BMI were, respectively, *M_weight_* = 76.51 kg (SD = 12.14), *M*_height_ = 157 cm (SD = 7.89), *M*_IMC_ = 3.92 (SD = 3.17). Baseline characteristics and dependent outcome measures are displayed in Table 1. Regarding sleep quality and fatigue, there were no significant differences between the groups. No adverse effects were indicated during the intervention, and adherence to the training programs was high (89.02% of the participants who started the intervention completed at least 83.33% of the sessions).

**Sleep Quality**

Post-intervention values and main effects regarding PSQI total score and domains are shown in Table 2. As for PSQI total score, our analysis revealed significant within-group differences for the HIIT group: *t*(25) = 2.28, *p* = 0.031, *d* = 0.44 and control group, *t*(21) = −3.663, *p* = 0.001, *d* = 0.28. The effect size for the PSQI total score of the HIIT group was small but close to moderate (d = 0.44), but this improvement was less than the minimal clinically important difference.

A detailed domain analysis of PSQI scores (Table 2) showed a decrease in subjective sleep quality between measurement points for the MIIT, *t*(22) =3.15, *p* =0.005, *d* =0.58, and HIIT groups, *t*(25) = 3.07, *p* = 0.005, *d* = 0.48. As for sleep latency, a statistically significant decrease became apparent for the HIIT between measurement points, *t*(25) = 2.10, *p* = 0.046, *d* = 0.40, whereas an increase occurred in the MIIT group, *t*(23) = 3.76, *p* = 0.001, *d* = 0.64. Additionally statistically significant differences were showed between the different groups in the post-intervention measure, *F*(2, 70) = 3.17, *p* = 0.048, *η*^2^ = 0.08 (medium effect). A posteriori comparisons using the Bonferroni correction showed that the HIIT and MIIT groups were statistically different from each other (-.753, *p* = 0.045, *d* = 0.70). Regarding the domain sleep duration, within-group differences were only observed for the MIIT group, *t*(23) = 2.94, *p* = 0.007, *d* = 0.61. The between-group ANOVA in the post-intervention measures was statistically significant, *F*(2, 70) = 3.73, *p* = 0.29, *η*^2^ = 0.10 (medium effect). A posteriori comparisons using the Bonferroni correction showed the existence of differences between the MIIT and control groups (−0.640, *p* = 0.040, *d* = 0.71. The analysis of the sleep disturbances domain showed an improvement for subjects in the HIIT group, *t*(23) = 3.14, *p* = 0.004, *d* = 0.67, as well as a worsening in the control group, *t*(22) = −2.79, *p* = 0.011, *d* = 0.45. In addition, differences were observed between groups in the post-intervention measure, *F*(2, 70) = 4.96, *p* = 0.01, *η*^2^ = 0.12 (medium effect). Specifically, the posterior comparisons using the Bonferroni correction reported the existence of statistically significant differences between the values observed in the HIIT and MIIT groups (−0.599, *p* = 0.009, *d* = 0.85).

Finally, no significant main effects were detected regarding the sleep efficiency and use of sleep medication domains, and the daytime dysfunction domain was only affected by a significant main effect as far as the time of measurement variable was concerned (Table 2).

**Fatigue**

**Figure 2:**
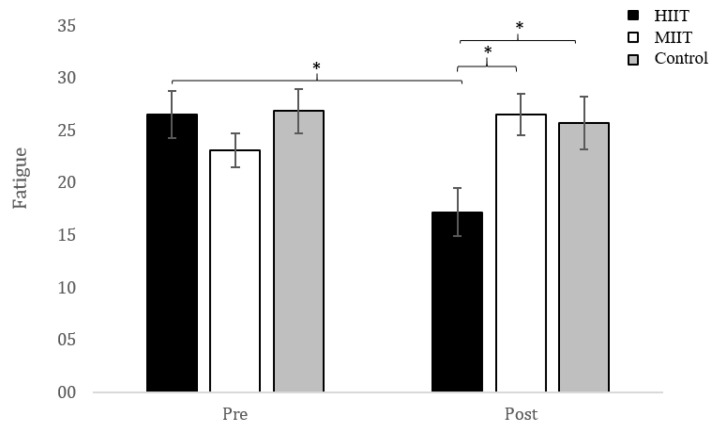
Pre- and post-intervention changes in FSS score. FSS: fatigue severity scale. HIIT: high-intensity interval training. MIIT: moderate-intensity interval training. * *p* < 0.05.

A statistically significant group x time interaction was obtained, *F*(2, 70) = 7.01, *p* = 7.01, *p* = 0.002, *η*^2^ = 0.17 (big effect). Neither the main effect of the group variable, *F*(2, 70) = 1.66, *p* = 0.198, nor the main effect of the measurement time variable, *F*(1, 70) = 2.73, *p* = 0.103, were statistically significant. The detailed analysis of the interaction showed the existence of significant differences between groups in the post-intervention measure, *F*(2, 70) = 5.38, *p* = 0.007, *η*^2^ = 0.133 (medium effect). A posteriori comparisons using the Bonferroni correction reported the existence of statistically significant differences between the HIIT and MIIT groups (−9.346, *p* = 0.013, *d* = 0.72), as well as HIIT and control (−8.498, *p* = 0.029, *d* = 0.87). In addition, only participants in the HIIT group experienced a significant decrease in their fatigue scores, *t*(25) = 3.32, *p* = 0.003, *d* = 0.80, between both measurement moments. This pre/post difference observed in the HIIT group (1.03) was larger than the minimal clinically important difference previously described (Figure 2).

## 4. Discussion

Our results obtained in this work can be considered novel, given the specific nature of the training program that the older adult population underwent. Our objective was to compare the effects of 12-week HIIT and MIIT programs with a TRX suspension system on sleep quality and fatigue. The findings showed that at post-intervention measurements, the HIIT group had experienced a decrease in fatigue levels. Regarding sleep quality, the HIIT group exhibited improvements in PSQI total score, sleep latency, sleep disturbances and subjective sleep quality. Notably, HIIT and MIIT had a good tolerance to exercise, obtaining physiological responses such as an adaptation to H during 12 weeks of training, being an important part as shown in other studies of interval training in the elderly, with positive results on physiological mechanisms [31,32,33].

Half of the older adult population reports difficulties in sleeping, which manifest in various ways such as reduced sleep efficiency, night awakenings, longer sleep onset, and more daytime naps [51]. Currently, different therapeutic methods have been used for its treatment, among which pharmaceutical treatment stands out. However, and given its side effects, other non-pharmacological interventions such as physical exercise have been recommended as the preferable option [52].

Sleep is fundamental to life, and its assessment provides essential health information; all sleep-assessment methods have benefits and disadvantages, although according to the study by Ibañez et al. [53], PSQI showed that its sensitivity is high to assess the quality of sleep As far as sleep quality is concerned, the analysis of PSQI global scores revealed a significant decrease between both measurements in the HIIT group. Karimi et al. [54] found similar results with scheduled exercises performed by a sample of older adults. After the intervention, the average score decreased in the training group (*p* = 0.001), with similar results to our high-intensity group, where our average score also decreased significantly (*p* = 0.006). However, unlike in our study, their sample suffered from primary insomnia, and their intervention only lasted eight weeks. In the subscales of the PSQI, sleep duration and sleep latency improved in our study for the MIIT group after the intervention period. Similarly, after a twelve-week yoga intervention in people over 60 years of age [55], and with a sample similar to ours in the subscales of sleep duration and sleep latency (*p* = 0.042 and *p* = 0.012), improvements were found in the same subscales. Poor scores in subjective sleep quality and sleep disturbances, as subscales of sleep quality (PSQI), decrease general health quality [4,5] among the elderly. Our findings showed post-intervention improvements in subjective sleep quality and sleep disturbances (*p* = 0.009 and *p* < 0.001), as did a Pilates intervention with a sample of elderly women for the same subscales of PSQI (*p* = 0.001 and *p* = 0.007) [56]. Contrary to our hypothesis, Bullock et al. [38] suggest that there may also be an intensity threshold for the impact of sleep-promoting exercise, such that high-intensity exercise could be harmful to sleep. Moreover, high-intensity exercise can cause increased muscle pain and physiological arousal, hampering the benefits of exercise on sleep rather than sleep. Fatigue is one of the foremost common complaints when seeking medical advice in medical care [57]. Our results showed that a 12-week HIIT training program improved fatigue in the elderly. However, the group that underwent the MIIT training showed no difference from the control group. Along equivalent lines, several studies have reported improvements in fatigue after differing types of exercise training. A recent study on older women showed that the practice of Pilates reduces feelings of fatigue after 12 weeks [56]. Another study [58] confirmed that physical exercise decreased fatigue in their participants, as did Jang et al. [59] when they examined the effects of foot reflexology on stress, fatigue, and even blood circulation, concluding that this intervention is effective in reducing such symptoms. Along similar lines, this study provides new data on the effects of other types of training, such as HIIT, on the fatigue experienced by the elderly.

The strengths of the present study include the use of a HIIT method based on suspension exercises, a novel approach that differs substantially from those commonly employed in the literature dealing with the health of older adults. Furthermore, another strength is its high degree of security as a training system. As a consideration, it is worth highlighting the importance of holding hands with TRX during the session. Some limitations must be acknowledged concerning this study: only short-term effects were measured, sleep quality was assessed subjectively and the preponderance of female participants. Future research should continue to expand along the lines established here, changing the type of exercise and defining long-term strategies to improve the health of elderly populations and their quality of life.

## 5. Conclusions

In conclusion, our study revealed that a population of elderly observed improvements in sleep quality and fatigue after a high-intensity intervention with a TRX suspension training system and the group moderate-intensity only observed improvements in subjective sleep domain after a TRX suspension training.

## Figures and Tables

**Table 1:** Baseline data of the participants.

		Total		HIIT		MIIT		Control		*p* Value
		(n = 73)		(n = 26)		(n = 24)		(n = 23)		
Age, years		68.49	(5.18)	68.23	(2.97)	68.75	(5.98)	68.52	(6.33)	0.940
Sex, %	Men	17	(100)	2	(11.8)	7	(41.2)	8	(47.1)	0.058
	Women	56	(100)	24	(42.9)	17	(30.4)	15	(26.8)	
Smoker, %	Yes	6	(100)	2	(33.33)	4	(66.67)	0	(0)	0.114
Weight, kg		76.51	12.15	73.75	14.20	76.48	12.09	79.65	9.03	0.240
Height, cm		157.00	7.89	155.65	7.60	159.13	8.12	15.637	7.85	0.266
BMI, kg/m^2^		30.593	3.25	2948	3.69	30.33	3.07	32.13	2.30	0.019
	No	67	(100)	2	(33.3)	4	(66.7)	0	(0)	
FSS		2.83	1.12	2.94	1.28	2.56	0.90	2.98	1.12	0.365
PSQI, score	Subjective sleep quality	1.18	(0.84)	1.23	(0.86)	1.38	(0.71)	0.91	(0.90)	0.156
	Sleep latency	1.30	(1.01)	1.46	(1.07)	1.13	(0.95)	1.30	(1.02)	0.506
	Sleep duration	1.18	(0.90)	1.15	(0.97)	1.21	(0.83)	1.17	(0.94)	0.978
	Sleep efficiency	1.08	(1.21)	0.92	(1.16)	1.25	(1.29)	1.09	(1.20)	0.640
	Sleep disturbances	1.55	(0.62)	1.69	(0.74)	1.58	(0.58)	1.35	(0.49)	0.148
	Use of sleeping medication	0.95	(1.32)	0.73	(1.19)	0.92	(1.35)	1.22	(1.44)	0.440
	Daytime dysfunction	0.51	(0.56)	0.50	(0.51)	0.38	(0.49)	0.65	(0.65)	0.234
	Total score	7.74	(4.31)	7.69	(3.93)	7.83	(4.45)	7.70	(4.75)	0.992
Poor sleep quality, %	Yes	46	(63.01)	17	(36.96)	15	(32.61)	14	(30.43)	0.946
	No	27	(36.99)	9	(33.33)	9	(33.33)	9	(33.33)	

Variables are expressed as frequencies (percentages) and mean (standard deviation). HIIT: high-intensity interval training. MIIT: moderate-intensity interval training. FSS: fatigue severity scale. PSQI: Pittsburgh sleep quality index.

**Table 2:** Effects of high- and moderate-intensity interval training programs (HIIT and MIIT) on sleep quality and fatigue.

		Post-Intervention Values														
		HIIT		MIIT		CONTROL (n = 23)		Group			Time			Group × Time		
		(n = 26)		(n = 24)												
		Mean	SD	Mean	SD	Mean	SD	*F* (2, 70)	*p*	*η* ^2^	*F* (1, 70)	*p*	*η* ^2^	*F* (2, 70)	*p*	*η* ^2^
FSS		1.91	1.30	2.95	1.07	2.85	1.34	1.66	0.198		2.70	0.103		7.01	0.002	0.17
PSQI	Subjective sleep quality ^†^	0.81	0.69	0.91	0.79	0.91	0.61	0.87	0.423		11.29	0.001	0.14	5.01	0.009	0.13
	Sleep latency	1.04	1.04	1.79	1.10	1.30	1.06	0.32	0.729		0.68	0.414		10.55	<0.001	0.23
	Sleep duration	0.81	0.80	0.71	0.81	1.35	0.98	1.11	0.335		4.81	0.032	0.06	3.85	0.026	0.10
	Sleep efficiency	0.69	0.93	1.13	1.30	1.35	1.30	1.15	0.322		0.06	0.808		1.31	0.277	
	Sleep disturbances	1.19	0.75	1.79	0.66	1.61	0.66	1.41	0.252		0.02	0.902		9.08	<0.001	0.21
	Sleep medication	0.92	1.29	1.17	1.43	1.35	1.30	0.88	0.421		1.99	0.162		0.06	0.421	
	Daytime dysfunction ^††^	0.62	0.75	0.67	0.58	0.96	0.71	2.28	0.111		6.16	0.016	0.08	0.43	0.654	
	Total score ^†††^	6.08	3.29	7.95	4.15	8.55	4.43	0.98	0.381		0.16	0.901		5.50	0.006	0.15

FSS = fatigue severity scale; HIIT= high-intensity interval training group; MIIT=moderate intensity interval training group; *η*^2^ = eta squared; PSQI= Pittsburgh sleep quality index; SD = standard deviation. ^†^ *F*(2, 68), since two outlier values observed in the post-intervention measure were not included in the analysis. ^††^ *F*(2, 67), since three outlier values observed in the post-intervention measure were not included in the analysis. ^†††^ *F*(2, 65), since five outlier values were not used for the calculation of the total score for sleep quality.

## Data Availability

The data presented in this study are available on request from the corresponding author. The data are not publicly available because, due to the sensitive nature of the questions asked in this study, participants were assured raw data would remain confidential and would not be shared.
